# Micro-structural Change During Nucleation: From Nucleus To Bicontinuous Morphology

**DOI:** 10.1038/srep15955

**Published:** 2015-11-03

**Authors:** Seongmin Jeong, Yongseok Jho, Xin Zhou

**Affiliations:** 1 Asia-Pacific Center for Theoretical Physics, Pohang, Gyeongbuk 790-784, South Korea; 2 Physics Department, POSTECH, Pohang, Gyeongbuk 790-784, South Korea; 3 School of Physical Science, University of Chinese Academy of Sciences, Beijing 100049, China

## Abstract

Although the microstructure of coexistence phase provides direct insights of the nucleation mechanism and their change is substantial in the phase transition, their study is limited due to the lack of suitable tools capturing the thermodynamically unstable transient states. We resolve this problem in computational study by introducing a generalized canonical ensemble simulation and investigate the morphological change of the nucleus during the water evaporation and condensation. We find that at very low pressure, where the transition is first order, classical nucleation theory holds approximately. A main nucleus is formed in the supersaturation near spinodal, and the overall shape of the nucleus is finite and compact. On increasing the pressure of the system, more nuclei are formed even before spinodal. They merge into a larger nuclei with a smaller free energy penalty to form ramified shapes. We suggest order parameters to describe the extent of fluctuation, and their relation to the free energy profile.

Nucleation is a key process that initiates phase transition or structural formation in nature^1^. Although it is often explained in the context of classical nucleation theory (CNT), numerous recent studies^2^,^3^,^4^,^5^,^6^,^7^,^8^,^9^,^10^,^11^,^12^,^13^,^14^,^15^,^16^,^17^,^18^ show that the nucleation processes are more complicated, commonly deviating from CNT to different extent, depending on the systems and conditions. These studies indicate that the microscopic detail of the intermediate states and especially their structures are the keys to the understanding nucleations and phase transitions. For this reason, imaging techniques are actively applied to observe metastable intermediate states, for example, colloidal crystallisation^2^,^17^, magnetite nucleation^13^,^14^, protein crystallisation on surface^15^,^16^, and crystalization of solutions^18^. These provide coarse insights into the nucleation mechanisms, such as one-step or multiple-step nucleations, single or multiple nucleation pathways, etc. However, in these experiments it is still difficult to capture the rare and short-lifetime transition states during phase transition and thus, the detailed mechanisms of nucleation, such as morphology of nuclei, are not well understood yet. For better understanding of nucleation, it is essential to observe these transient intermediate nuclei, especially the successive change of nuclei in shape, size and structure.

Conventional numerical simulations could not detect these unstable transient states. Instead, these researches focus on the high supersaturation regions near the spinodals (or stability limits), where transitions may occur within the simulation accessed time scale^19^. For low supersaturations close to binodals (or equal probability phases), the transition is inaccessible by the standard simulation methods in finite simulation time. Indirect simulation techniques^20^,^21^ have been developed to construct the free energy profiles along preselected reaction coordinates and to estimate the kinetics of nucleation.

In this work, we extend the accessible states of simulations to the whole intermediate regions between two phases. For this purpose, we develop a new simulation strategy, the generalized canonical ensemble (GCE) simulations combined with the replica exchange molecular dynamics (REMD) techniques^22^, which enables us to sufficiently sample over the whole conformational space, not only the stable phases and metastable supersaturated phases, but also the unstable coexistent phases in canonical ensemble. Throughout, we can obtain an integrated view of the microscopic mechanisms of nucleation.

We apply the GCE-REMD to water evaporation and condensation which are the most frequently encountered phase transition in daily life, but still not well understood. The result of a Lennard-Jones system is also presented as comparison. Most previous computational studies on water evaporation focused on supersaturations region, up to spinodals^23^,^24^,^25^,^26^,^27^,^28^. The coexistence regions between spinodals, which are directly related to the microscopic mechanisms of the phase transitions, have been revealed yet. Our work fills the gap between these regimes and show that the morphologies of coexistence in both water and LJ are sensitive to the modulation in the pressure. At very low pressures, the coexistence is in agreement with the assumption of CNT, the shape of the major nucleus is sphere-like in the supersaturation region nearby spinodal. On increasing the pressure of the system, more nuclei are formed even in low supersaturation region far away from the spinodal. They merge into each other to form ramified shapes. The fluctuating nucleus stretches to the whole simulation box at high pressures. It would develop a real decomposition at the critical point.

## Results

We perform GCE-REMD simulations under a constant pressure constraint for both the Lennard-Jones(LJ) fluid and a coarse-grained water model, the mW model^29^. The number of particles *N* is 250 for both the systems. We also simulate in larger systems (*N* = 1000) at a few typical pressures in order to check the finite-size effects. Sufficiently high exchange rate through the replicas ensures the equilibrium in our simulations in the whole enthalpy space, including the stable gas/liquid phases, the metastable supercooled gas and superheated liquid, as well as the gas-liquid phase coexistences, therefore the samplings satisfy the expected equilibrium distribution of the GCE, which can convert to the equilibrium distribution in the normal canonical ensemble (Fig. S1, S2). The GCE-REMD simulation sufficiently samples at each iso-enthalpy subspace, and thus it provides the most probable equilibrium intermediates during the phase transition, which give the transition pathways at least for lower supersaturation. More details about the simulations and the relation of the GCE to the normal canonical ensemble are presented in the method section.

**Figure 1:**
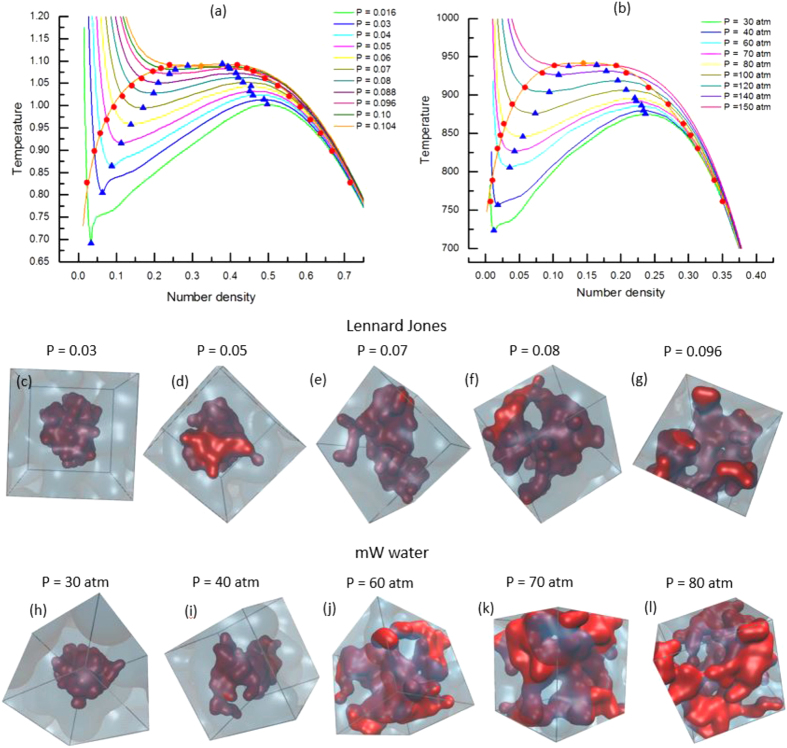
Equations of state from GCE-REMD are plotted for (**a**) LJ and (**b**) mW water system. Liquid-vapor binodals and spinodals are plotted as red circles and blue triangles, respectively. The critical points are measured as 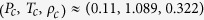 for LJ, and 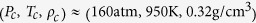 for mW water. In bottom panls, the representative snapshots at the phase-coexistence are displyed for both LJ and mW water at different pressures.

Figure 1 illustrates the central findings of the paper. In (a), we plot 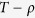 curves for LJ system 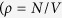 is a number density). The pressure varies from *P* = 0.016 to 0.104 near the critical point (dimensionless unit). We estimate the critical point as 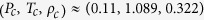. The results agree well with the referential estimates^30^,^31^,^32^. In (b) we present 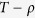 curves for the mW water. In the work, we choose one of the simplistic water model, mW water^29^, in order to reach equilibrium in the whole enthalpy regions by the GCE-REMD simulation within a reasonable computational cost. The mW model is known to be quantitively not very accurate, but captures all the essential physical properties. This is good enough for our purpose to capture the microscopic structure of liquid at different pressures of water. The critical point of mW water is found at 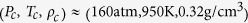.

In the bottom panels of Fig. 1, we plot the representative snapshots of LJ and mW water at the enthalpies which correspond to the coexistence temperatures, *i. e.*, the temperatures in which the number of liquid particles equals to that of gas particles at different pressures. The snapshots are sampled without any bias inside each intermediate iso-enthalpy subspace, thus they are regarded as typical intermediates during the phase transition. It clearly shows that the micro-structure of the intermediates changes from nucleus to bicontinuous coexistence on increasing pressure. At very low pressure (such as *P* = 0.03 in LJ, or *P* = 30atm in the mW), the curves represent the first order phase transition. During the phase transition, the densities at two phases are significantly different. The interfacial energy is large enough to confine the minor phase within a finite volume and the interface deforms a little from the spherical surface. On increasing pressure, the interface between two phases becomes rough and the minor phase has a branched structure. At high pressure, densities at gas and liquid spinodals converge. The interfacial energy becomes negligible and the thermal fluctuations are dominant, which leads to the inter-connected continuous interfaces.

To analyze this quantitatively, we divide the simulation configurations into liquid clusters and gas clusters based on the Voronoi cell analysis: a particle is identified as liquid-like if its Voronoi cell volume is less than a threshold volume, and it is gas-like otherwise. The threshold volume is determined by analyzing the distribution of Voronoi cell volume in both the liquid and gas phases (Fig. S3). Particles are grouped into a neighbourhood if they share a common Voronoi cell surface. In this way, we can classify all the particles as liquid or gas clusters (Fig. S4). All the interfacial Voronoi cell surfaces between liquid and gas particles form the interface between liquid and gas phases. The area of the interface is obtained by simply summing up all the areas of the interfaces between two phases.

**Figure 2:**
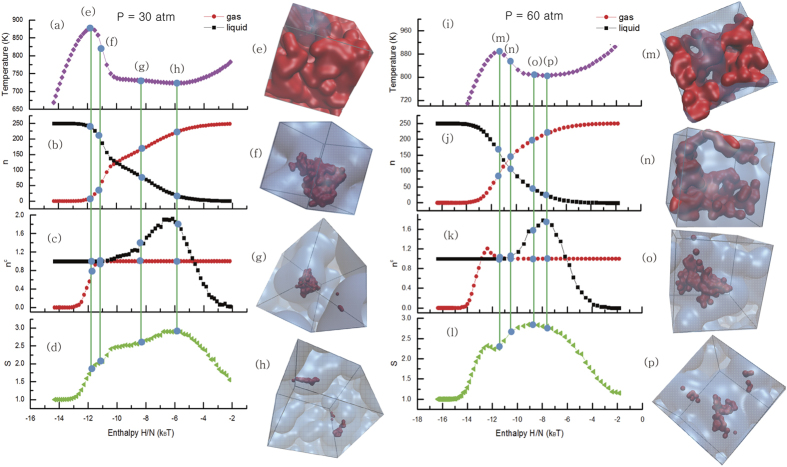
In the mW water system, the statistical temperature, the size of maximal droplet and bubble, the number of droplets and bubbles, the shape factor of liquid-gas interface, as well as some typical snapshots at different enthalpy are shown at the low pressure *P* = 30atm (the left panels) and at the high pressure *P* = 60atm (the right panels). These snapshots are chosen from four enthalpy regions, as shown in the order parameter curves, which corresponding to liquid spinodal (**e**,**m**), liquid droplet (**f**,**n**), bubble (**g**,**o**), and gas spinodal (**h**,**p**), respectively. The red circles and black squares in (**b**,**c**,**j**,**k**) correspond to that of gas bubble and liquid droplet, respectively.

In Fig. 2, we plot the order parameters, (a) the statistical temperature 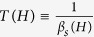, (b) the size of largest liquid and gas cluster, 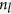 and 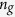, respectively, (c) the number of liquid and gas clusters, 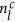 and 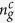, respectively, (d) shape factor *s*, as a function of the enthalpy of per particle. Here


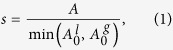


where *A* is the total area of all the interfacial Voronoi cell planes between liquid and gas clusters, and 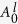 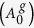 is the area of the sphere which has the same volume of total liquid phase (the gas phase). The results at low pressure are shown in the left panel. At low enthalpy, tiny gas bubbles start to form at superheating liquid. At liquid spinodal (e), a dominant gas bubble starts to grow gradually, as in (b) and (c), and their shape factor is still small. After the spinodal (f), only one bubble remains and quickly grows. Its shape is now more rough. On the opposite direction of the pathway (condensation), more liquid droplets are formed around the gas spinodal. The number of droplets already is reduced to one even at the supercooled gas region. Contrary to the gas bubbles, there exist a few small liquid droplets which are disconnected from the major liquid droplet. This is because bubbles are easier to be connected than droplets.

The mechanism is very different at higher pressure, 60atm, which is still far lower than the critical pressure, 160atm, as seen in Fig. 2 (i)~(p). The range of the phase coexistence region is thinner than that at low pressure (i). Small seeds are created at earlier stage of the phase transition while the phase is still gas or liquid (j) and (k). It implies that less supersaturation is required for the small seeds to evolve to a large cluster due to the fluctuations. At the high pressure, the surface tension is small due to the smaller difference in density between gas and liquid, so, less energy is exerted in the coalescence of a cluster. The low interfacial tension also contributes to the interface being very flexible and rough, then the two phase are continuously inter-connected to each other to form the biocontinuous phase (n). The results in LJ are found very similar to that in the mW qualitatively. For checking the possible finite-size effects, we also simulate the larger systems (*N* = 1000) for both the LJ and mW water (Figs S6–S9), the results are very similar to that in the small systems. At low pressures, the coexistent phase looks like a deformed spherical nucleus. At high pressures, more complex morphologies are found. Although there is a little difference in quantity, but we can’t find any substantial difference to invalidate our conclusion. In fact, this larger system results show this trend in *S*, and 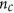 more clearly, and strengthen our conclusion.

**Figure 3:**
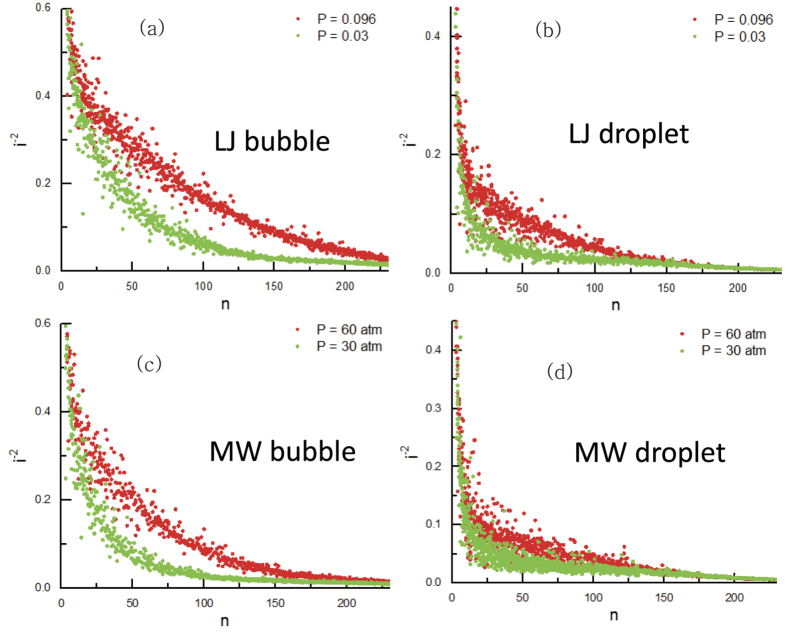
Connectivity of different size gas bubbles and liquid droplets for the LJ system (**a,b**), and for the mW water system (**c,d**), respectively. Red circles for high pressure results, and the green circles for low pressure results.

We consider the connectivity in the largest cluster, which is a measure of the number of neighbouring particles inside a cluster. In the network-like structure there exist a few large connections in the core, but small connections in threads. Besides, in the bulk-like structure large connections are dominant. To contrast small connections we use 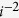 instead of the neighbouring number *i* itself. The connectivity is formally expressed as,


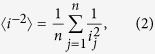


where *n* is the number of particles in the largest liquid (or gas) cluster, *j* is the index of the particles inside the largest cluster, and 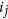 is the number of neighbours of particle *j* inside cluster. In Fig. 3, we plot 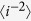 of the different-size liquid droplets and bubbles. For all cases, 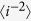 at high pressure is significantly larger than that at low pressure. It provides a clearer evidence that the shape of the liquid (or gas) cluster at high pressure is always more rough than that at low pressure in the whole enthalpy region. Especially, the difference of 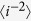 is kept significantly larger through the bicontinuous region, where both liquid and gas make a single cluster 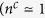, and 50 ≲ n_g_ ≲ 125, as Fig. 2(b,c,j,k).

## Discussion

Although the enthalpy may not be a perfect reaction coordinate of the phase transition, it can be a good approximation. So the sampled conformations in the GCE-REMD with successive enthalpies provide an approximate pathway of the nucleation process. In addition, the simulation is unbiased on each iso-*H* subspace, and thus the pathway of nucleation (the morphological change along the realistic reaction coordinate *x*) can be constructed along the enthalpy. Therefore, the structural change at coexistence during the gas-liquid phase transition of the mW water and LJ systems at various pressures provides the mechanism of the phase transitions. At very low reduced pressure, the phase transition is followed by CNT or its slight modification, since the interfacial energy is large enough to confine the minor phase into a finite size nucleus. Here the reduced pressure means the relative pressure to the critical value. On increasing pressure, the interfacial energy is reduced. The thermal fluctuations easily overcome the deforming energy of the surface of nuclei and the nucleation gradually deviates from the CNT. At high enough reduced pressure, whose value depends on systems, the fluctuation length exceeds the periodic domain size of the system and the system develops a bicontinuous morphology. We believe that this continuously transit to the real decomposition at the critical point.

Our findings on the morphology, beyond the spinodals and its dependence on pressure encompassing the first order to near second order phase transition, provide an integrated view in the kinetics of the nucleation process. Since the connectivity of the nucleus is finite in the first order phase transition, but diverges in second order phase transition, it also can be served as an indicator to distinguish between the first order and the second order phase transition. Because, our results prove that the free energy cost forming nucleus should strongly depend on the the microscopic structure of the nucleus, it will lead to the development of new theories of nucleation and first order phase transition that conisders the microstructure of the nucleus.

## Methods

### Generalized canonical ensembles replica exchange simulations

In the GCE simulations, the number of particles, and the volume (or here pressure) of system are fixed as the canonical ensemble, but the temperature is expressed as a linear function of the enthalpy *H*,


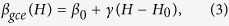


where, 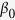, *γ*, and 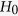 are the control parameters. Here we fix the pressure *P*. 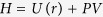 is the enthalpy with the potential energy 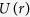, and volume *V*. Thermodynamic stability is judged by 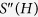 which is the second order derivative of the micro-canonical entropy,


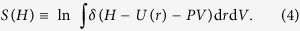


For *H* that satisfies 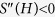, the system is stable thermodynamically, and accessible by conventional CE. However, it is unstable and can’t be accessed by CE where 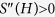. In gas-liquid phase transition, gas, liquid and supersaturations satisfy 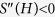, but 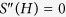 at spinodals, and turns to 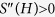 at coexistence states. So, conventional CE can’t access the spinodals, and coexistence states. In the GCE, we can resolve this problem by choosing a large enough positive *γ* in the coexistence region, which satisfies


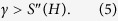


Within this approach, we access the spinodals and the intermediate states.

In thermodynamics, the GCE at fixed pressure is equivalent to the NPT ensemble by introducing an effective potential,


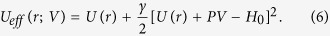


Its conformational distribution is


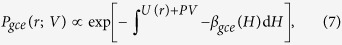


and thus we can keep the conventional MC or MD scheme with the effective potential. Technically, here the GCE is implemented as the standard NPT molecular dynamics simulation by scaling the total physical force of each particle by the factor 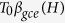 with the thermostat temperature 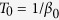.

By varying the set of parameters, the GCE can detect any enthalpy region, including the stable liquid/gas regions, the metastable supersaturation region, and the coexistence region. However, similar to the standard canonical ensemble (CE) simulations, a single GCE simulation might not be easy to be ergodic within a finite simulation time, the enhanced sampling techniques developed in the CE can be applied to the GCE to shorten the equilibrium time of simulations. Here we have incorporated the replica exchange molecular dynamics (REMD) method to form the GCE-REMD. The implementation of the GCE-REMD is direct^22^, similar to the standard REMD. More details about GCE-REMD are presented in Supplemental Material (SM).

### Reproduce equilibrium properties from GCE-REMD

The GCE may look similar to the umbrella sampling simulation, and so does the GCE-REMD to the umbrella sampling replica exchange^33^. However, we emphasize that there exist substantial differences in the physics. In the umbrella sampling, usually a very large spring coefficient, *γ*, is applied to constraint simulation around the center of spring 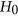, and the canonical average is reproduced by sample reweighing techniques^34^ from all the replicas. In contrast, in the GCE-REMD, any *γ*, that satisfies 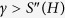, stabilizes the coexistence states. The averages can be directly related to that of CE by estimating the microscopic entropy, through


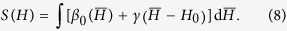


Here 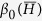 is the reverse function of the average enthalpy obtained in the GCE simulations, 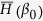, under the fixed 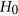 and *γ*.

For any order parameter, *x*, the free energy profile at a special temperature *T* is obtained from the GCE-REMD simulations,


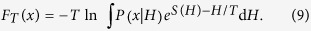


Here the conditional probability, 
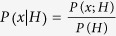
, is an intrinsic property of the system, which is independent of the GCE parameters, and so does the microscopic entropy 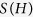. Therefore, although both 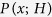 and 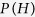 which are the probabilities to find a sample at 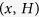 and at *H* in the GCE-REMD simulation, respectively, depend on the selection of parameters in the simulations, 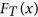 is independent on these parameters. If *x* is the *H* itself, we have, 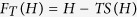. It *x* is properly chosen as a real reaction coordinate, 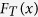 provides significant understanding to the phase transition. Since the selection of *x* is independent of the GCE simulation, it does not bring any possible bias in simulation to understand the nucleation as in previous methods^20^,^21^.

### Simulation details

In this work, we focus on the morphological change of coexistence in the mW water (and LJ) by varying the enthalpy. We simulate systems with two different number of particles, the smaller one has 250 particles, and the larger one has 1000 particles. The detailed simulation parameters are listed as Table S1 and S2 in SM. At atmospheric pressure, the statistical temperature in the mW water drops very sharply right after the liquid spinodal, and thus the exchange between replicas is barely accepted. At the lowest pressure we can reach equilibrium within the reasonable simulation time scale (4 × 10^7^ time steps) by 64 replicas is about 30atm for the smaller mW water system. To check the dependence on the initial conditions, the simulations start from two different initial conformations (liquid and gas), and at equilibrium we obtain the same results regardless of the initial conditions. In the larger mW system, we only simulate at two pressures 30 and 60 atm for checking the possible finite-size effects. In both cases, we slightly decrease the enthalpy range of simulations to make the enthalpy overlapping between neighbouring replicas not very small thus the simulation can reaches the equilibrium within tens million MD steps. We also have checked the possible effects of the cutoff distance of the interaction by varying 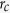 from 2.5 to 3.5 in the LJ systems (Fig. S7). As we have presented the results in SM, we obtain similar results including morphology of coexistence, except a shift of critical temperature as expectation (It is well known that the change of cutoff distance for small system may lead quantitative shift although the difference is not substantial.). More details are presented in SM.

## Additional Information

**How to cite this article**: Jeong, S. *et al.* Micro-structural Change During Nucleation: From Nucleus To Bicontinuous Morphology. *Sci. Rep.* **5**, 15955; doi: 10.1038/srep15955 (2015).

## Supplementary Material

Supplementary Information
