# Supplementary material for: Micro-structural Change During Nucleation: From Nucleus To Bicontinuous Morphology
Source: Sci Rep. 2015 Nov 3;5:15955. doi: 10.1038/srep15955 (PMC4630657; doi:10.1038/srep15955)
Supplement: Supplementary Information [file srep15955-s1.pdf]

Supplementary Materials of "Micro-structural Change During Nucleation: From Nucleus To Bicontinuous Morphology"

SeongMin Jeong, YongSeok Jho, and Xin Zhou

A. Generalized Canonical Ensemble Replica Exchange MD (GCE-REMD)

The GCE is same as the normal isothermal-isobaric (NPT) ensemble but replace the original potential energy *U(r)* by the effective potential $U_{eff}(r)$,

$$U_{eff}(r) = U(r) + \frac{\gamma}{2}\{U(r) + PV - H_0\}^2 \quad \text{(S1)}$$

where, r is a simple notation of the configuration coordinate of N particles, $\gamma > 0$, and H_0 are control parameters, *P* is the external control constant pressure, *V* is the volume of configuration.

Replica exchange simulation within GCE is similar to that in the normal canonical ensemble (CE) with the effective potential. The acceptance criterion of exchange configurations between replicas can be directly derived same as that in CE by detailed balance condition. The acceptance probability of exchanging configurations of mth and n^{th} replica is,

$$\mathrm{acc}(\mathrm{m},\mathrm{n}) = \min\{1, \exp[\Delta]\}.$$

In replicas of GCE-REMD, we use the same pressure P and temperature $T = 1/\beta$ but different parameter $H_0^{(m)}$. Thus Δ is described by,

$$\Delta = \beta\gamma\left(H_0^{(n)} - H_0^{(m)}\right)\left(H^{(n)} - H^{(m)}\right). \quad \text{(S2)}$$

Here $H^{(m)}$ is the enthalpy $H = U + PV$ in replica m. In the present work, we only consider the exchange between nearest neighbours, *i.e.* $\mathrm{n} = \mathrm{m} + 1$, for simplicity.

B. Simulation detail

We performed a replica exchange molecular dynamics simulations within isothermal-isobaric GCE for 250 Lennard-Jones particles system under the periodic boundary condition. At initial, we perform short simulations for optimization, which consists of 5 step cooling down processes (50000 MD step each) from T_i^* to target thermostat temperature T_f^*. All input parameters are presented at the table1 (Lennard-Jones) and table2 (mW water). GCE-REMD simulation consists of the M replicas with a fixed pressure P and M uniform-separated. $H_0^{(j)}(1 \leq \mathrm{j} \leq \mathrm{M})$ between H_0^i and H_0^f. H_0^i and H_0^f are lower and upper limit of the H_0 , respectively. All replicas start from the same initial configurations at either gas or liquid phase. The replica exchange was carried out simultaneously for each adjacent pairs in two possible sets, $\left\{\left(H_0^{(1)}, H_0^{(2)}\right), \left(H_0^{(3)}, H_0^{(4)}\right), \ldots, \left(H_0^{(M-1)}, H_0^{(M)}\right)\right\}$ and $\left\{\left(H_0^{(2)}, H_0^{(3)}\right), \left(H_0^{(4)}, H_0^{(5)}\right), \ldots, \left(H_0^{(M-2)}, H_0^{(M-1)}\right)\right\}$. The set of pairs for replica exchange is randomly selected per every 100 MD steps. The data for enthalpy and configurations are collected per every

1000 and 10000 MD steps.

B1) Lennard-Jones Liquid

Lennard-Jones (LJ) interaction energy U(r) is given by

$$\mathrm{U(r)} = \begin{cases} U_{LJ}(r) - U_{LJ}(r_c) & r < r_c \\ 0 & r \geq r_c \end{cases},$$

where $U_{LJ}(r) = 4\epsilon(\sigma^{12}/r^{12} - \sigma^6/r^6)$ is the full LJ interaction, ε and σ are the well depth and diameter of the LJ particle, and $r_c = 2.5\sigma$ is the cutoff distance. All quantities are rescaled by characteristic length σ, mass m and energy ϵ, *i.e.*, potential energy $U^* = U\epsilon^{-1}$, pressure $P^* = P\sigma^3\epsilon^{-1}$, density $\rho^* = \rho\sigma^3$, time $\Delta t^* = \left(\sigma\sqrt{(m/\epsilon)}\right)^{-1} t$ and temperature $T^* = k_B T\epsilon^{-1}$. For convenience, we omit the asterisk symbol of reduced quantities.

B2) mW Water

mW water model consists of two parts; radial interaction for inter atomic interaction and angular interaction which results in the hydrogen bonded structure.

$$\mathrm{E} = \sum_i \sum_{j>i} \varphi_2(r_{ij}) + \sum_i \sum_{j\neq i} \sum_{k>j} \varphi_3(r_{ij}, r_{ik}, \theta_{ijk})$$

$$\varphi_2(r) = A\varepsilon\left[B\left(\frac{\sigma}{r}\right)^p - \left(\frac{\sigma}{r}\right)^q\right] exp\left(\frac{\sigma}{r - a\sigma}\right)$$

$$\varphi_3(r, s, \theta) = \lambda\varepsilon[cos\theta - cos\theta_0]^2 exp\left(\frac{\xi\sigma}{r - a\sigma}\right) exp\left(\frac{\xi\sigma}{s - a\sigma}\right)$$

where A=7.049556277, B=0.6022245584, p=4, q=0, and $\xi = 1.2$. The reduced cutoff distance is $\mathrm{a} = 1.8$ and $\theta_0 = 109.47°$ ensures tetrahedral structure. In the current work, we scaled the distance and energy unit with $\sigma = 2.3925\text{Å}$ and $\varepsilon = 6.189\mathrm{Kcal/mol}$. The $\lambda = 23.15$ was selected which is known to reproduce actual water's vaporization enthalpy well. The initial number density is fixed as 0.0008 for all 5 different pressures.

B3) Simulation parameters

Table S1. Simulation parameters of GCE-REMD in Lennard-Jones fluids

P^*	N	r_c^*	Δt^*	$\mathrm{N}_{MD}(10^6)$	H_0^i	H_0^f	M	γ
0.016	250	2.5	0.002	76.2	100.00	-1300.00	64	0.010
0.030	250	2.5	0.002	59.6	-50.00	-1600.00	100	0.006
0.040	250	2.5	0.002	20.3	100.00	-1235.36	60	0.006
0.050	250	2.5	0.002	27.5	70.00	-1239.00	60	0.006
0.060	250	2.5	0.002	20.4	70.00	-1239.00	60	0.006
0.070	250	2.5	0.002	27.6	50.00	-1241.00	60	0.006
0.080	250	2.5	0.002	118.3	50.49	-1241.14	60	0.006

0.088	250	2.5	0.002	29.0	70.00	-1239.00	60	0.006
0.096	250	2.5	0.002	37.3	-200.00	-1350.00	60	0.006
0.100	250	2.5	0.002	66.7	50.00	-1239.36	64	0.006
0.104	250	2.5	0.002	51.8	1.00	-1250.00	60	0.006
0.030	1000	2.5	0.002	51.3	400.00	-5000.00	60	0.006
0.096	1000	2.5	0.002	68.6	-1100.00	-1910.36	60	0.006
0.096	1000	3.5	0.002	62.9	-800.00	-3500.00	60	0.006

Table S2. Simulation parameters of GCE0REMD for mW water

P(atm)	N	Δt(fs)	$N_{MD}(10^6)$	H_0^i	H_0^f	M	γ
30	250	5.0	49.3	-1.00	-300.00	64	0.010
40	250	5.0	24.9	-1.00	-300.00	64	0.010
60	250	5.0	39.3	150.00	-400.00	64	0.003
70	250	5.0	29.3	200.00	-400.00	64	0.003
80	250	5.0	13.9	200.00	-400.00	64	0.003
100	250	5.0	20.1	150.00	-400.00	64	0.003
120	250	5.0	22.6	150.00	-400.00	64	0.003
140	250	5.0	55.1	150.00	-400.00	64	0.003
150	250	5.0	15.3	-1.00	-300.00	60	0.010
30	1000	5.0	30.3	-1.00	-1200.00	64	0.010
60	1000	5.0	19.0	200.00	-720.00	64	0.001

C. Efficiency of isobaric-isothermal GCE-REMD

In order to compare the efficiency of the standard replica exchange (stREMD) and GCE-REMD, we simulate LJ system with $N = 250$, $P = 0.08$, with $M = 60$ replicas. We used equally divided thermostat temperature T between 0.7 and 1.2 for stREMD. For GCE-REMD, we equally divided H_0 between 50.49 and -1241 while fixing T at 3.0 and $\gamma = 0.006$. Figure S1 displays the temperature of the replicas in terms of (a) enthalpy and (b) number density for stREMD and GCE-REMD, respectively.

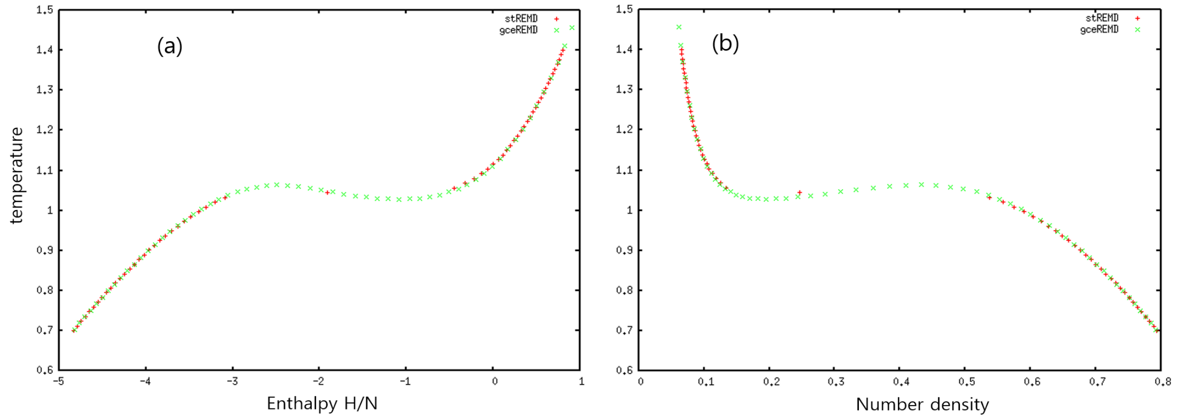

Figure S1. (a) Temperatures T(H) in terms of enthalpy H/N and (b) Temperatures $T(\rho)$ in terms of number density at pressure P = 0.08 by stREMD (red cross) and GCE-REMD (green cross). In gas and liquid phases, the results in GCE are same as that in CE. Our results verify those.

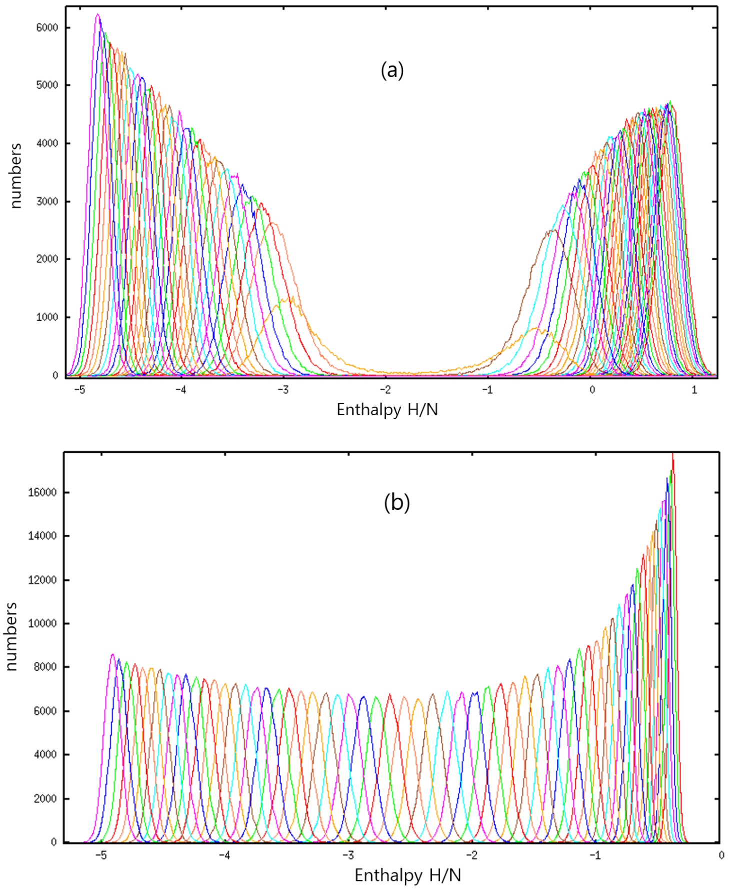

Figure S2. The Lennard-Jones enthalpy histograms of replica 1,2,…,60 at pressure P = 0.008 by stREMD and GCE-REMD respectively. In stREMD, there is a gap region without sufficient visiting, which corresponding to the gas/liquid coexistence. Due to the exist of the gap, the suitable temperature setting is difficult to get sufficient exchange rates. In GCE-REMD, the whole the enthalpy space is equally visited, thus the replica exchange has more efficient like simulation within the single liquid/gas phase.

D. Analysis Details

D1) Identification of liquid and gas. We use the Voronoi cell volume of a particle to identify it as liquid-like or gas-like particle. The neighbouring same-like particles form clusters of liquid or gas.

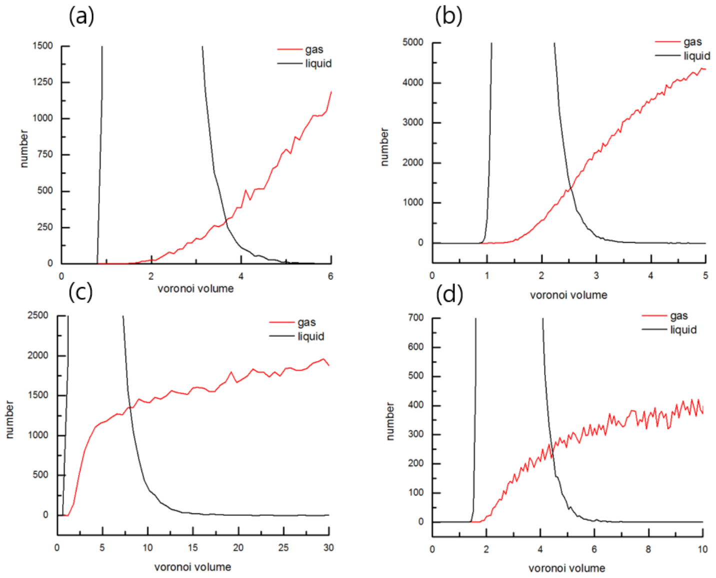

Figure S3. The histograms of voronoi volume for (a) P = 0.03 atm, (b) P = 0.096 in Lennard Jones system and (c) P = 30 atm, (d) P = 60 atm in mW water system respectively. The red and black colored solid line show the pure gas and liquid phase data. The histogram data are obtained by obvious gas and liquid phase configurations. The each cross points for gas and liquid are (a) 3.7, (b) 2.6, (c) 8.1 and (d) 4.5. These values are used to identify the gas and liquid phase.

D2) While counting the number of liquid/gas clusters, in the main text, we only take into account larger clusters which include number of particles more than a threshold (usually 2 or 3). As a comparison, here we also count all the clusters even a cluster only involves one particle.

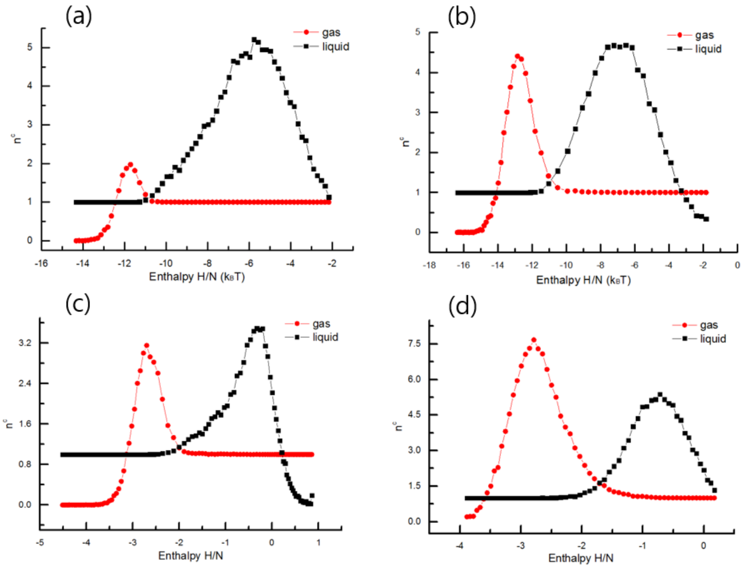

Figure S4. The total number of clusters (n_c) including single-particle clusters. (a), (b), (c), and (d) are at P = 30 atm, P = 60 atm in mw water system, and P = 0.03, P = 0.096 in Lennard Jones system, respectively.

D3) Order parameters in LJ, (N=250)

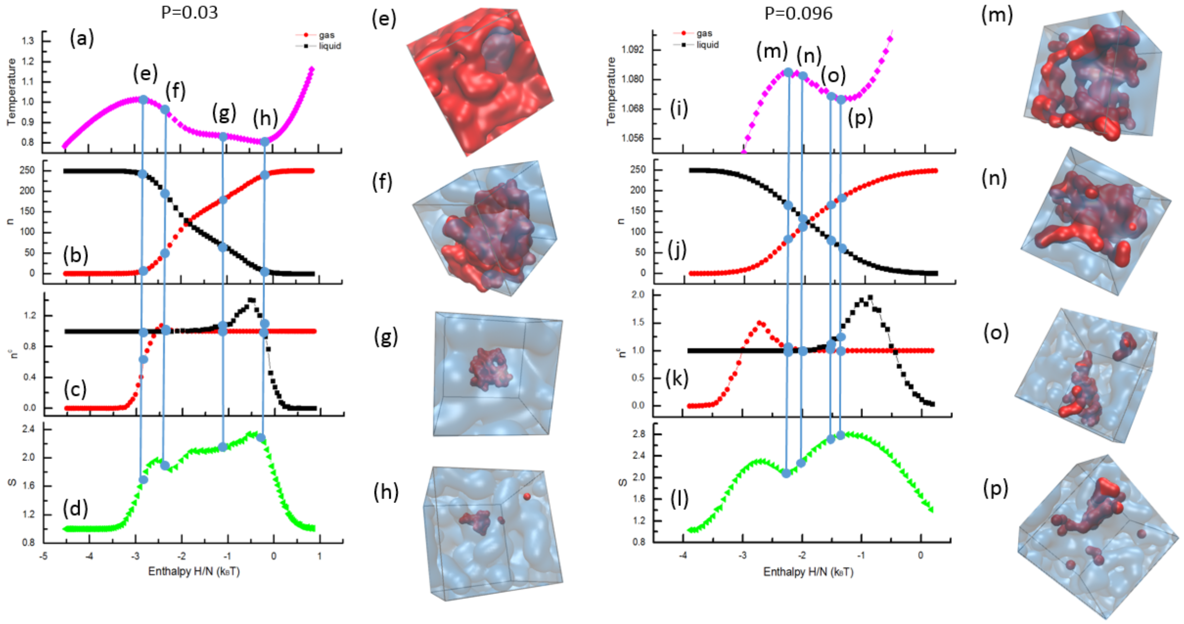

Figure S5. In the LJ system, the statistical temperature, the size of maximal droplet and bubble, the number of droplet s and bubbles, the shape factor of liquid-gas interface, as well as representative snapshots at different enthalpy are shown at the low pressure P=0.03 (the left panels) and at the high pressure P=0.096 (the right panels) . These snapshots are chosen from four enthalpy regions, as shown in the order parameter curves, which correspond to liquid spinodal (e) and (m), liquid droplet (f) and (n), bubble (g) and (o), and gas spinodal (h) and (p), respectively. The red circles and black squares in (b), (c), (j) and (k) correspond to that of gas bubble and liquid droplet, respectively.

E. Large system results (N=1000)

E1) Order parameters in LJ

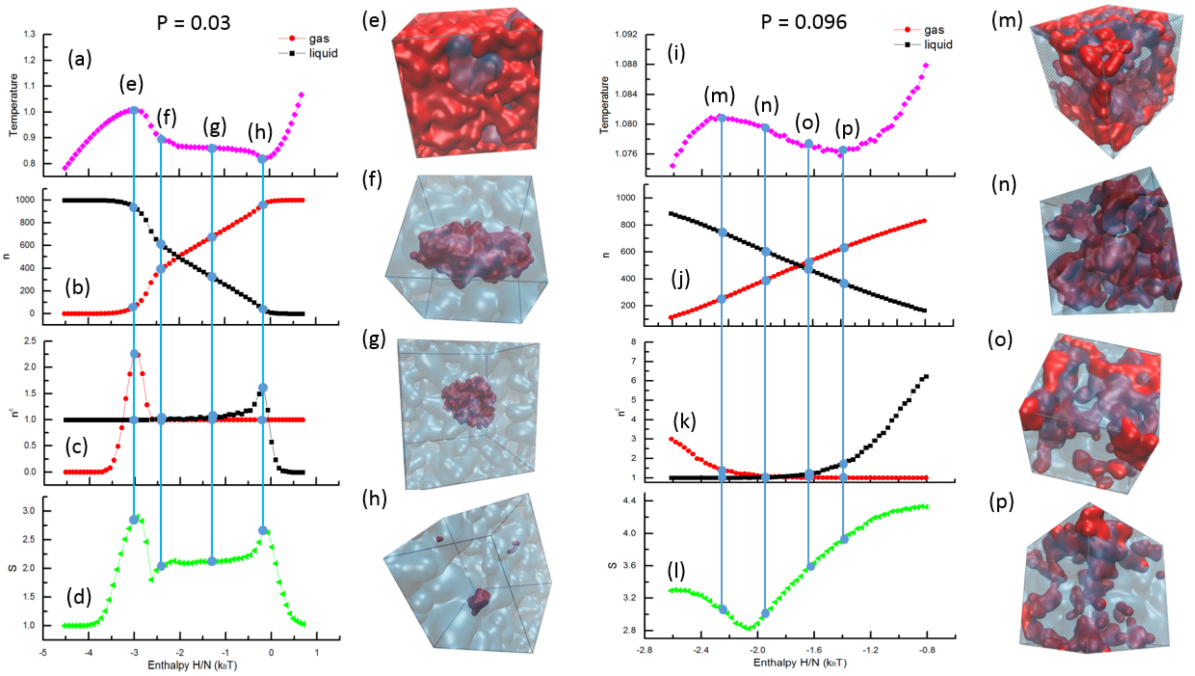

Figure S6. In the N=1000 LJ system, the statistical temperature, the size of maximal droplet and bubble, the number of droplets and bubbles, the shape factor of liquid-gas interface, as well as representative snapshots at different enthalpy are shown at the low pressure P = 0.03 (the left panels) and at the high pressure P = 0.096 (the right panels). These snapshots are chosen from four enthalpy regions, as shown in the order parameter curves, which correspond to liquid spinodal (e) and (m), liquid droplet (f) and (n), bubble (g) and (o), and gas spinodal (h) and (p) respectively. The red circles and black squares in (b), (c), (j) and (k) correspond to that of gas bubble and liquid droplet, respectively. There are small deviations in quantities between N = 250 LJ results (S5) and N = 1000 LJ results. Nevertheless the difference is not substantial. There exist distinct increments of n^c, S for higher pressure, which in fact strengthens our main conclusion.

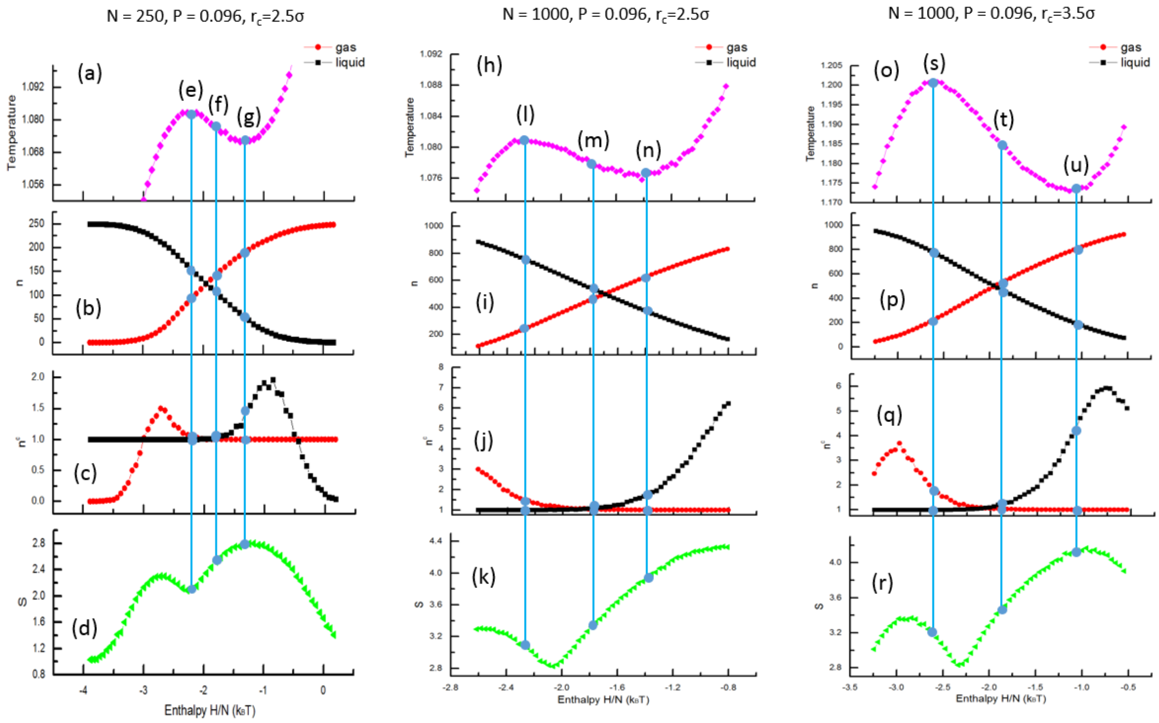

Figure S7. The order parameters of LJ system at high pressure $\mathrm{P} = 0.096$ for $\mathrm{N} = 250, r_c = 2.5\sigma$ (left panels), $\mathrm{N} = 1000, r_c = 2.5\sigma$ (middle panels) and $\mathrm{N} = 1000, r_c = 3.5\sigma$ (right panels) are plotted. In the middle panels and right panels, the cutoff distance is different while the rest conditions are remained the same. Although there exist a small quantitative difference (which is well known), there is no substantial difference with respect to r_c. There is a little deviation in the size of the number of droplets and bubbles n^c.

E2) Order parameters of mW water

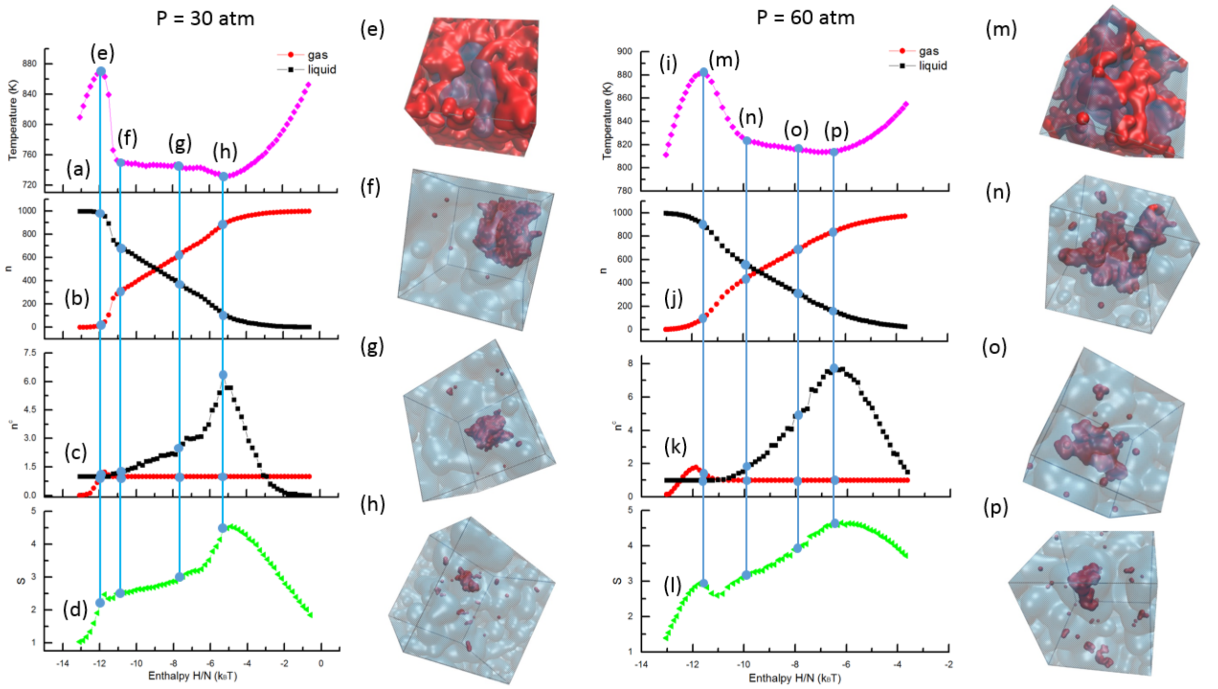

Figure S8. In the N=1000 mW water system, the statistical temperature, the size of maximal droplet and bubble, the number of droplets and bubbles, the shape factor of liquid-gas interface, as well as representative snapshots at different enthalpy are shown at the low pressure P = 30 atm (the left panels) and at the high pressure P = 60 atm (the right panels). These snapshots are chosen from four enthalpy regions, as shown in the order parameter curves, which correspond to liquid spinodal (e) and (m), liquid droplet (f) and (n), bubble (g) and (o), and gas spinodal (h) and (p) respectively. The red circles and black squares in (b), (c), (j) and (k) correspond to that of gas bubble and liquid droplet, respectively. The whole results are consistent with N=250 mW water results (Figure 2). There exist distinct increments of n^c, S for higher pressure, which in fact strengthens our main conclusion.

E3) Connectivity of different size gas bubble and liquid droplets

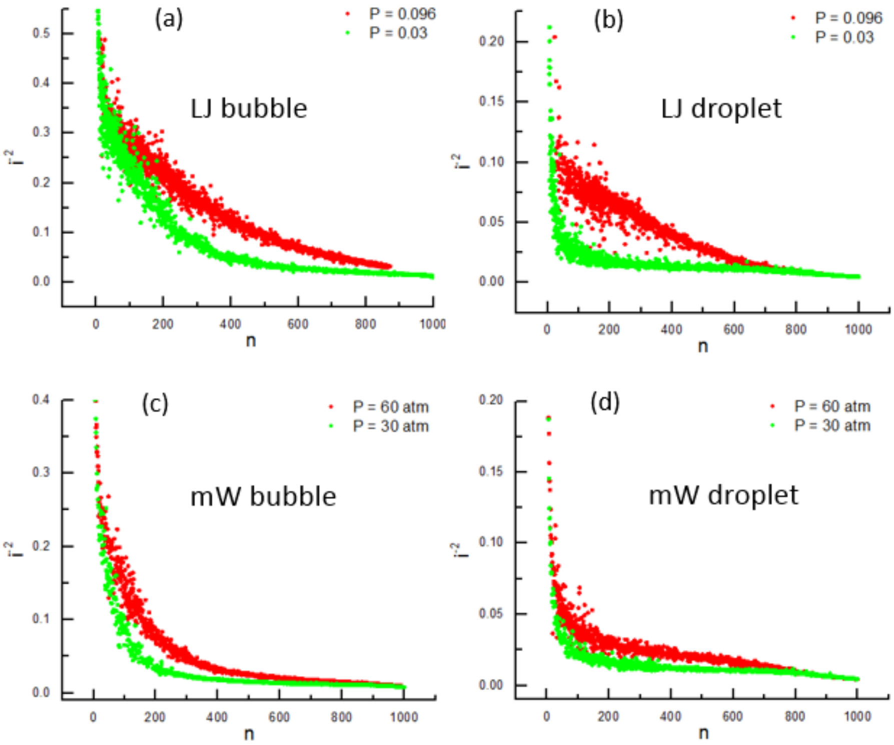

Figure S9. Connectivity of different size gas bubbles and liquid droplets for the $N = 1000$ LJ system (a) and (b), and for the $N = 1000$ mW water system (c) and (d), respectively. Red circles for high pressure results, and the green circles for low pressure results.
